# Relapse of both small cell lung cancer and Lambert–Eaton myasthenic syndrome after a 13-year disease-free survival period

**DOI:** 10.1186/s40880-016-0127-x

**Published:** 2016-07-02

**Authors:** Fumio Asano, Keisuke Watanabe, Masaharu Shinkai, Yoshitaka Tei, Kei Mishina, Mikiko Tanabe, Hiroshi Ishii, Masahiro Shinoda, Tadasuke Shimokawaji, Makoto Kudo, Takeshi Kaneko

**Affiliations:** Respiratory Disease Center, Yokohama City University Medical Center, 4-57 Urafune-cho, Minami-ku, Yokohama, Kanagawa 232-0024 Japan; Department of Pathology, Yokohama City University Medical Center, 4-57 Urafune-cho, Minami-ku, Yokohama, Kanagawa 232-0024 Japan; Comprehensive Cancer Center, Yokohama City University Medical Center, 4-57 Urafune-cho, Minami-ku, Yokohama, Kanagawa 232-0024 Japan; Department of Pulmonology, Yokohama City University Graduate School of Medicine, 3-9 Fukuura, Kanazawa-ku, Yokohama, Kanagawa 236-0004 Japan; Department of Medicine, McMaster University, Hamilton, ON Canada

**Keywords:** Lambert–Eaton myasthenic syndrome, Paraneoplastic syndrome, Paraneoplastic neurological syndrome, P/Q-type anti-voltage-gated calcium channel antibody, Small cell lung carcinoma

## Abstract

Lambert–Eaton myasthenic syndrome (LEMS) is a paraneoplastic syndrome and only 3% of small cell lung carcinoma (SCLC) patients have LEMS. Moreover, the recurrence of SCLC after a disease-free survival (DFS) of more than 10 years is rare. We report a patient who had a recurrence of both SCLC and LEMS after a 13-year DFS period. A 69-year-old man was diagnosed with LEMS and SCLC (cT0N2M0, stage IIIA) 13 years ago. Chemoradiotherapy was performed and a complete response was achieved. With anticancer treatment, the LEMS symptoms was alleviated. At the age of 82 years, gait disturbance appeared followed by left supraclavicular lymphadenopathy and further examination revealed the recurrence of SCLC. Careful screening for the recurrence of SCLC might be needed when the patient has recurrent or secondary paraneoplastic neurological syndrome even after a long DFS period.

## Background

Lambert–Eaton myasthenic syndrome (LEMS) is a paraneoplastic syndrome and only 3% of small cell lung carcinoma (SCLC) patients have LEMS [1]. In addition, SCLC recurrence after a disease-free survival (DFS) of more than 10 years is rare [2–4]. Herein, we report a patient with a recurrence of both SCLC and LEMS after a 13-year DFS period. Our case might indicate the importance of SCLC recurrence screening in patients with a recurrence or a secondary paraneoplastic neurological syndrome (PNS) even after a long DFS period.

## Case presentation

**Fig. 1:**
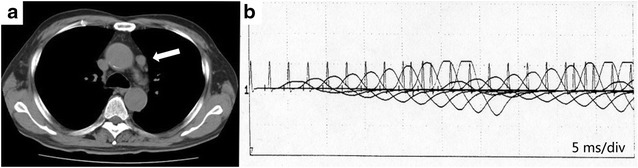
Radiological findings and electromyography on a small cell lung carcinoma (SCLC) patient with Lambert–Eaton myasthenic syndrome (LEMS). **a** Chest computed tomography (CT) revealed mediastinal lymphadenopathy (*arrow*) on February 7, 2002. **b** Electromyography following stimulation at 50 Hz showed waxing on March 13, 2002

**Fig. 2:**
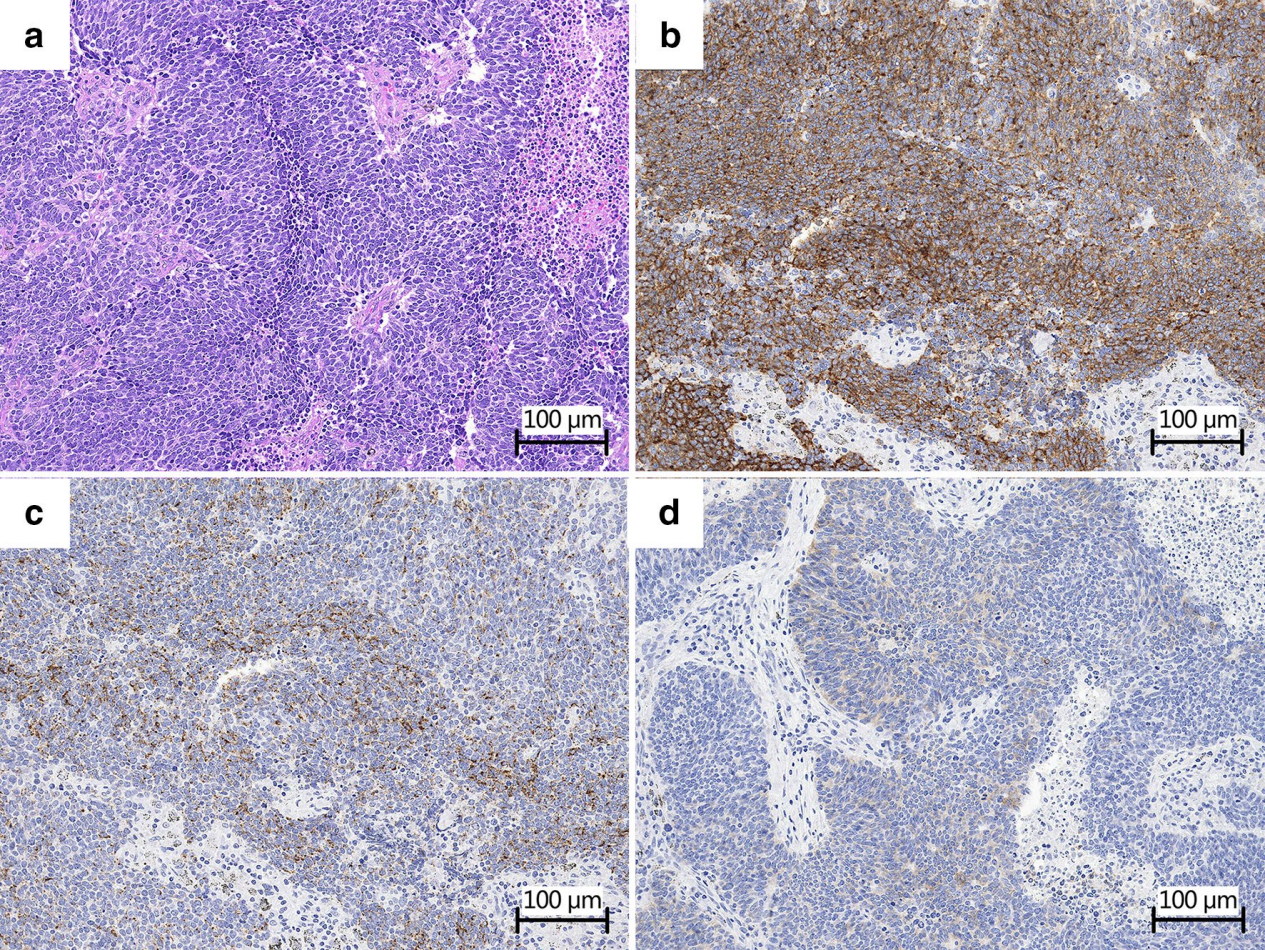
Pathological and cytological findings in an SCLC patient with LEMS. Specimens were obtained by cervical lymph node biopsy on February 14, 2002. Paraffin-embedded sections were stained with antibody using the polymer method (The Dako Envision™ FLEX, Dako Japan, Tokyo, Japan) and signal was developed with diaminobenzidine (DAB). Sections were counterstained with hematoxylin. Positive staining for markers in cytoplasm and on cell membrane was indicated in *brown*. **a** There are dense sheets of small cells with nuclear molding and necrosis (hematoxylin–eosin staining). **b** CD56 was diffusely and strongly positive on cell membrane. **c** Chromogranin A was diffusely and moderately expressed in cytoplasm. **d** Synaptophysin was focally and weakly expressed in cytoplasm

In December 2001, a 69-year-old man was referred to our hospital for gait disturbance and dysarthria. Chest computed tomography (CT) showed mediastinal lymphadenopathy (Fig. 1a) and the accumulation of 2-deoxy-2-[18F]fluoro-d-glucose (FDG) was detected by positron emission tomography (PET). The serum level of pro-gastrin-releasing peptide was 38.3 pg/mL. The specimen obtained by lymph node biopsy with mediastinoscopy revealed small cell carcinoma (Fig. 2) and he was diagnosed with SCLC (cT0N2M0, stage IIIA). The serum level of P/Q-type anti-voltage-gated calcium channel (VGCC) antibody was extremely high (1476 pmol/L; upper limit of normal, 20 pmol/L) and electromyography (EMG) showed waxing at 50 Hz (Fig. 1b). Therefore, the diagnosis of LEMS with SCLC was made. Plasmapheresis, intravenous immunoglobulin and 3,4-diaminopyridine therapy were performed, but the symptoms did not improve and the serum level of P/Q-type VGCC antibody remained high (1608 pmol/L).

Carboplatin (area under the curve: 5 mg/mL, day 1) and etoposide (100 mg/m^2^, days 1–3) were intravenously administered every 4 weeks (total eight cycles) and radiotherapy of the mediastinum with a total dose of 45 Gy (1.5 Gy/fraction, twice daily) was delivered. A complete response was achieved. The serum level of P/Q-type VGCC antibody had remarkably decreased to 958 pmol/L after the first three chemotherapy cycles; after all eight cycles, it decreased from 1608 to 40 pmol/L. Gait disturbance and dysarthria were alleviated. SCLC had not recurred for 13 years.

**Fig. 3:**
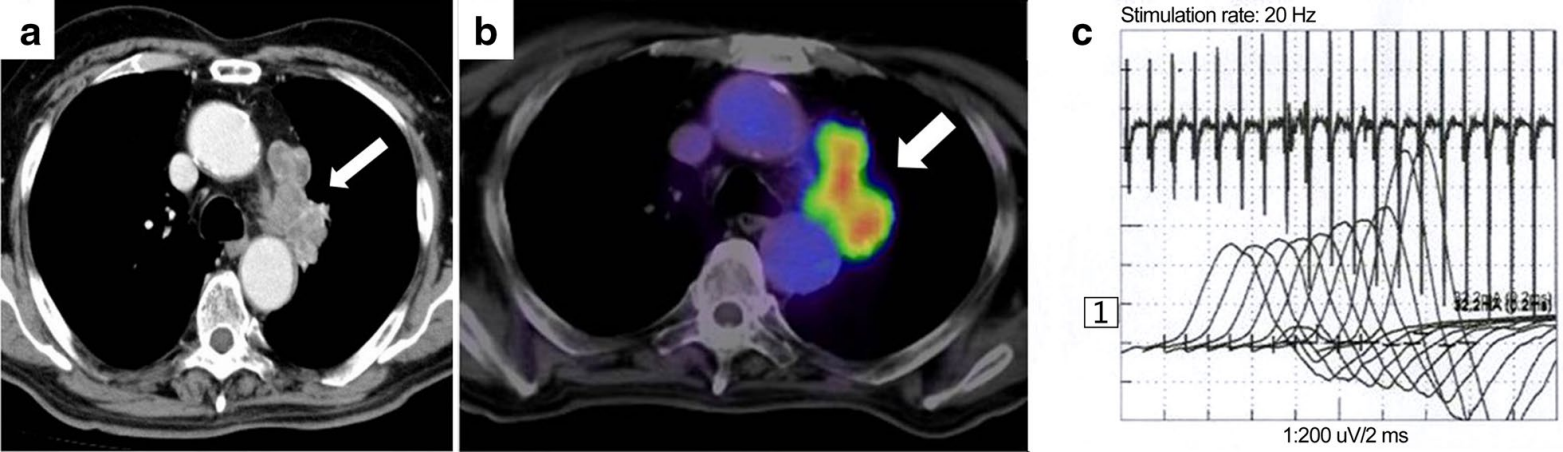
Radiological findings and electromyography in the SCLC patient with LEMS at the age of 85 years. **a** Chest contrast CT revealed mediastinal and pulmonary hilar lymphadenopathy (*arrow*) on March 10, 2015. B, 2-deoxy-2-[18F]fluoro-d-glucose (FDG) positron emission tomography (PET)-CT showed FDG accumulation in the mediastinal and pulmonary hilar lymph nodes (*arrow*) on March 24, 2015. **c** Electromyography following stimulation at 20 Hz showed waxing on April 9, 2015

**Fig. 4:**
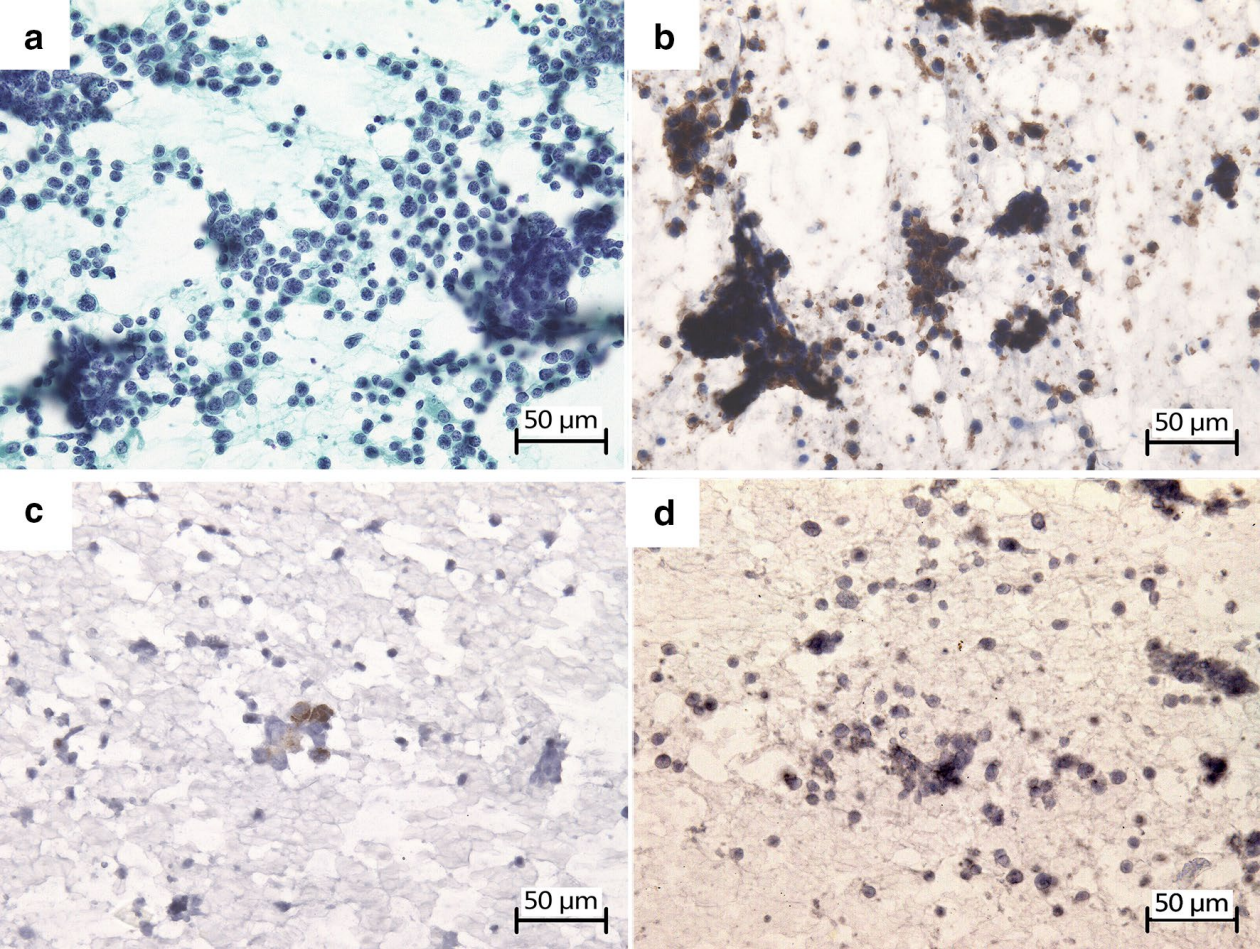
Cytological findings in the SCLC patient with LEMS at the age of 85 years. Specimens were obtained by cervical lymph node needle aspiration biopsy on March 3, 2015. Paraffin-embedded sections were stained with antibody using the polymer method and signal was developed with DAB. Sections were counterstained with hematoxylin. Positive staining for markers in cytoplasm and on cell membrane was indicated in *brown*. **a** Cytological examination revealed small cell carcinoma. **b** CD56 was diffusely and strongly positive on cell membrane. **c** Chromogranin A was focally and moderately expressed in cytoplasm. **d** Synaptophysin was not expressed in cytoplasm

In January 2015, at the age of 82 years, the patient developed gait disturbance followed by left supraclavicular lymphadenopathy. Further examination of left supraclavicular lymphadenopathy was performed. Physical examination revealed proximal muscle weakness and reduced tendon reflexes in the lower extremities. His chest radiography showed a 3.5 × 2.5 cm nodule on the aortic arch of the left upper lung lobe and a 4.4 × 2.3 cm nodule on the pulmonary hilar of the left middle lung lobe. Chest contrast CT revealed supraclavicular, mediastinal and pulmonary hilar lymphadenopathy (Fig. 3a) and FDG PET-CT showed FDG accumulation (Fig. 3b). The serum level of pro-gastrin-releasing peptide was 442.3 pg/mL. Cervical lymph node needle aspiration biopsy revealed small cell carcinoma in the supraclavicular lymph node (Fig. 4) and the patient was diagnosed with SCLC recurrence. The serum level of P/Q-type VGCC antibody was 172.6 pmol/L and EMG showed waxing at 20 Hz (Fig. 3c). Therefore, he had recurrence of both SCLC and LEMS. Carboplatin (area under the curve: 5 mg/mL, day 1) and etoposide (100 mg/m^2^, days 1–3) were intravenously administered every 4 weeks (a total of six cycles and the dose was reduced to 80% from the fourth cycle because of grade 4 neutropenia). Chemotherapy achieved a partial response and alleviation of his symptoms.

## Discussion

When lung cancer is diagnosed after a long DFS period, it is difficult to decide whether it is due to a recurrence or a secondary primary lung cancer. A 5-year DFS is an indicator of the cure of SCLC [5]. However, in some cases of SCLC, a recurrence after a DFS period of more than 10 years has been reported [2–4]. We considered the present case as a recurrence of SCLC for a few reasons. First, the immunological staining pattern of specimens obtained in 2015 was similar to that in 2001, although synaptophysin expression was not observed in 2015. Second, recurrence is more common than a second primary SCLC in SCLC patients with a long survival period. In 60 patients with SCLC who survived for more than 5 years, nine had SCLC recurrence and none had a second primary SCLC [6]. Johnson et al. [7] studied 62 SCLC patients with a DFS period of more than 2 years and reported that 18 had recurrence whereas two had a second primary SCLC. Third, considering the doubling time, a recurrence after a DFS period of more than 13 years is quite possible [2]. Moreover, in our case, SCLC reappeared on the same side of the mediastinal lymph nodes. Therefore, we considered this case as a recurrence of SCLC. A small amount of SCLC cells had grown for 13 years. However, there remains the possibility of recurrence.

In the study of secondary PNS by Ducray et al. [8], it was reported that, in all patients with a cancer recurrence, a secondary PNS antedated the cancer recurrence. In our case, gait disturbance was presented before the diagnosis of an SCLC recurrence. If the patient has a recurrence or a secondary PNS, careful screening for cancer recurrence is needed.

The etiology of PNS recurrence might be mediated by onconeural antibody production stimulated by the recurrence of SCLC. In this case, the serum level of P/Q-type VGCC antibody was elevated with the recurrence of SCLC and LEMS. A previous study reported that, among the patients with PNS positive for onconeural antibody, the same antibody was detected when a secondary PNS appeared [8]. Nagashima et al. [9] reported an SCLC patient with anti-Hu antibody who experienced a recurrence of SCLC with a secondary PNS. However, in contrast to our cases, those patients had a secondary PNS different from the first one [8, 9]. Anti-Hu antibody is related to some types of PNS, such as sensory neuropathy, cerebellar ataxia and limbic encephalitis [10]. On the other hand, P/Q-type VGCC antibody was relatively specific to LEMS. The antibody was detected in 85–90% of patients with LEMS [11] and 10 of 12 SCLC patients with the P/Q-type VGCC antibody had LEMS [1]. This might be owing to the difference between our case and the cases reported in literature. It is unclear how the type of PNS was determined, although the same antibody was detected. Further study is needed to understand the etiology of PNS.

## Conclusions

Recurrence of both SCLC and LEMS after a DFS period of more than 10 years is rare. However, careful screening for cancer recurrence is needed if the patient has a recurrence or a secondary PNS.

## Consent

Written informed consent was obtained from the patients for publication of this case report and any accompanying image.
